# 3-Chloro­quinuclidinium chloride

**DOI:** 10.1107/S1600536808011434

**Published:** 2008-04-26

**Authors:** Isha Azizul, Arifin Zainudin, Seik Weng Ng

**Affiliations:** a Department of Chemistry, University of Malaya, 50603 Kuala Lumpur, Malaysia

## Abstract

The cation of the title compound, C_7_H_13_ClN^+^·Cl^−^, forms a linear hydrogen bond to the chloride anion. The cation is disordered about a mirror plane.

## Related literature

For isomeric 4-chloro­quinuclidinium chloride, see: Kurahashi *et al.* (1980 ▶), which also reports the parent quinuclidinium chloride.
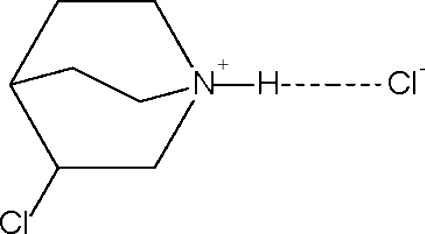


## Experimental

### 

#### Crystal data

C_7_H_13_ClN^+^·Cl^−^
*M* *_r_* = 182.10
Orthorhombic, 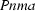

*a* = 9.379 (1) Å
*b* = 8.067 (1) Å
*c* = 11.482 (2) Å
*V* = 868.7 (2) Å^3^
*Z* = 4
Mo *K*α radiation
μ = 0.68 mm^−1^
*T* = 100 (2) K
0.15 × 0.08 × 0.03 mm

#### Data collection

Bruker SMART APEX diffractometer
Absorption correction: multi-scan (*SADABS*; Sheldrick, 1996 ▶) *T*_min_ = 0.872, *T*_max_ = 1.000 (expected range = 0.855–0.980)
5307 measured reflections
1068 independent reflections
856 reflections with *I* > 2σ(*I*)
*R*_int_ = 0.047

#### Refinement

*R*[*F* ^2^ > 2σ(*F* ^2^)] = 0.039
*wR*(*F* ^2^) = 0.113
*S* = 1.02
1068 reflections
82 parameters
58 restraints
H atoms treated by a mixture of independent and constrained refinement
Δρ_max_ = 0.31 e Å^−3^
Δρ_min_ = −0.57 e Å^−3^

### 

Data collection: *APEX2* (Bruker, 2007 ▶); cell refinement: *SAINT* (Bruker, 2007 ▶); data reduction: *SAINT*; program(s) used to solve structure: *SHELXS97* (Sheldrick, 2008 ▶); program(s) used to refine structure: *SHELXL97* (Sheldrick, 2008 ▶); molecular graphics: *X-SEED* (Barbour, 2001 ▶); software used to prepare material for publication: *publCIF* (Westrip, 2008 ▶).

## Supplementary Material

Crystal structure: contains datablocks global, I. DOI: 10.1107/S1600536808011434/bq2068sup1.cif

Structure factors: contains datablocks I. DOI: 10.1107/S1600536808011434/bq2068Isup2.hkl

Additional supplementary materials:  crystallographic information; 3D view; checkCIF report

## Figures and Tables

**Table 1:** Hydrogen-bond geometry (Å, °)

*D*—H⋯*A*	*D*—H	H⋯*A*	*D*⋯*A*	*D*—H⋯*A*
N1—H1⋯Cl1	0.88 (1)	2.13 (1)	3.008 (3)	175 (4)

**Fig. 1.**
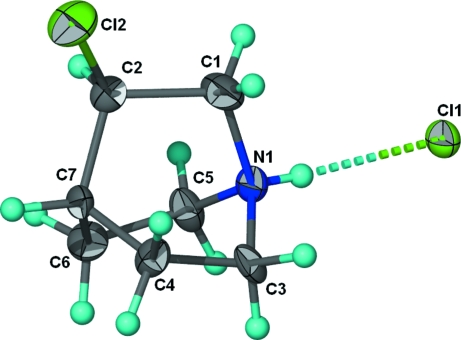
Thermal ellipsoid plot of the two independent molecules of 2-chloroquinuclidinium chloride at the 70% probability level; hydrogen atoms are drawn as spheres of arbitrary radius. The dashed lines denote the hydrogen bond.

## supplementary crystallographic information

### Comment

4-Chloroquinuclidinium chloride features an N–H···Cl hydrogen bond between the cation and anion. The N–C and C–C bonds are somewhat shorter than those in the unsubstituted salt, and this has been attributed to the electron-withdrawing effect of the chlorine substituent (Kurahashi *et al.*, 1980). The present isomeric compound (Scheme I) is expected to show this feature; however, owing to disorder, the effect cannot be unambiguously observed even at low temperature. The cation forms a linear hydrogen bond [N–H···Cl 3.008 (3) Å] to the chloride; the cation is disordered about a mirror plane (Fig. 1).

### Experimental

The commercially available compound was a crystalline. A large block was cut into a smaller specimen.

### Refinement

The cation is disordered about a mirror plane in the carbon atoms except C1 atom. The N1 and C1 atoms, which lie on this symmetry element, were refined with their normal half occupancies. The other carbon atoms were refined with half occupancies, subject to N–C being restrained to 1.49±0.01 Å and C–C to 1.54±0.01 Å. Additionally, the 1,3-related distances were restrained from 2.43±0.01 Å, to 2.47±0.01 Å as well as 2.52±-0.01 Å. The anisotropic temperature factors of the disordered carbon were restrained to be nearly isotropic but the N–H distance was restrained to 0.88±0.01 Å.

Carbon-bound H-atoms were placed in calculated positions (C—H 0.99 to 1.00 Å) and were included in the refinement in the riding model approximation, with *U*(H) set to 1.2*U*(C). The ammonium H-atom was located in a difference Fourier map, and was refined with an N–H distance restraint of 0.88±0.01 Å; its temperature factor was freely refined.

### Crystal data

**Table:**

C_7_H_13_ClN^+^·Cl^–^	*F*_000_ = 392
*M**_r_* = 182.10	*D*_x_ = 1.408 Mg m^−^^3^
Orthorhombic, *P**n**m**a*	Mo *K*α radiation λ = 0.71073 Å
Hall symbol: -P 2ac 2n	Cell parameters from 909 reflections
*a* = 9.379 (1) Å	θ = 3.1–22.9º
*b* = 8.067 (1) Å	µ = 0.68 mm^−^^1^
*c* = 11.482 (2) Å	*T* = 100 (2) K
*V* = 868.7 (2) Å^3^	Block, colorless
*Z* = 4	0.15 × 0.08 × 0.03 mm

### Data collection

**Table:**

Bruker SMART APEX diffractometer	1068 independent reflections
Radiation source: fine-focus sealed tube	856 reflections with *I* > 2σ(*I*)
Monochromator: graphite	*R*_int_ = 0.047
*T* = 100(2) K	θ_max_ = 27.5º
φ and ω scans	θ_min_ = 2.8º
Absorption correction: multi-scan(SADABS; Sheldrick, 1996)	*h* = −12→8
*T*_min_ = 0.872, *T*_max_ = 1.000	*k* = −10→10
5307 measured reflections	*l* = −14→13

### Refinement

**Table:**

Refinement on *F*^2^	Secondary atom site location: difference Fourier map
Least-squares matrix: full	Hydrogen site location: inferred from neighbouring sites
*R*[*F*^2^ > 2σ(*F*^2^)] = 0.039	H atoms treated by a mixture of independent and constrained refinement
*wR*(*F*^2^) = 0.113	*w* = 1/[σ^2^(*F*_o_^2^) + (0.0562*P*)^2^ + 0.8408*P*] where *P* = (*F*_o_^2^ + 2*F*_c_^2^)/3
*S* = 1.02	(Δ/σ)_max_ = 0.001
1068 reflections	Δρ_max_ = 0.31 e Å^−^^3^
82 parameters	Δρ_min_ = −0.57 e Å^−^^3^
58 restraints	Extinction correction: none
Primary atom site location: structure-invariant direct methods	

### Fractional atomic coordinates and isotropic or equivalent isotropic displacement parameters (Å^2^)

**Table:**

	*x*	*y*	*z*	*U*_iso_*/*U*_eq_	Occ. (<1)
Cl1	0.69230 (8)	0.2500	0.57828 (7)	0.0218 (2)	
Cl2	0.10483 (11)	0.2500	0.30411 (8)	0.0374 (3)	
N1	0.3718 (3)	0.2500	0.5682 (2)	0.0190 (6)	
H1	0.4648 (12)	0.2500	0.576 (3)	0.035 (12)*	
C1	0.3382 (3)	0.2500	0.4416 (3)	0.0331 (9)	
H1A	0.4092	0.1832	0.3983	0.040*	0.50
H1B	0.3397	0.3645	0.4106	0.040*	0.50
C2	0.1846 (4)	0.1722 (5)	0.4282 (3)	0.0184 (9)	0.50
H2	0.1947	0.0495	0.4194	0.022*	
C3	0.2957 (7)	0.3924 (13)	0.6253 (9)	0.0204 (17)	0.50
H3A	0.3397	0.4988	0.6019	0.024*	0.50
H3B	0.3013	0.3825	0.7111	0.024*	0.50
C4	0.1402 (5)	0.3855 (6)	0.5851 (5)	0.0212 (11)	0.50
H4A	0.1248	0.4653	0.5209	0.025*	0.50
H4B	0.0763	0.4159	0.6503	0.025*	0.50
C5	0.3285 (7)	0.0917 (13)	0.6251 (10)	0.024 (2)	0.50
H5A	0.3698	0.0857	0.7043	0.029*	0.50
H5B	0.3651	−0.0034	0.5795	0.029*	0.50
C6	0.1650 (5)	0.0823 (7)	0.6325 (4)	0.0244 (12)	0.50
H6A	0.1323	0.1111	0.7120	0.029*	0.50
H6B	0.1316	−0.0310	0.6139	0.029*	0.50
C7	0.1058 (4)	0.2079 (5)	0.5430 (4)	0.0205 (12)	0.50
H7	0.0007	0.1931	0.5330	0.025*	

### Atomic displacement parameters (Å^2^)

**Table:**

	*U*^11^	*U*^22^	*U*^33^	*U*^12^	*U*^13^	*U*^23^
Cl1	0.0157 (4)	0.0284 (4)	0.0212 (4)	0.000	−0.0006 (3)	0.000
Cl2	0.0340 (5)	0.0546 (6)	0.0236 (5)	0.000	−0.0111 (4)	0.000
N1	0.0145 (13)	0.0241 (13)	0.0184 (14)	0.000	−0.0020 (10)	0.000
C1	0.0176 (17)	0.064 (3)	0.0182 (19)	0.000	0.0006 (13)	0.000
C2	0.019 (2)	0.0171 (19)	0.019 (2)	0.0018 (17)	−0.0032 (16)	0.0013 (17)
C3	0.019 (3)	0.017 (3)	0.025 (3)	−0.004 (3)	0.008 (3)	0.004 (2)
C4	0.015 (2)	0.022 (3)	0.026 (3)	0.004 (2)	−0.004 (2)	−0.006 (2)
C5	0.017 (3)	0.019 (3)	0.036 (4)	0.000 (3)	0.007 (3)	−0.001 (3)
C6	0.028 (3)	0.026 (3)	0.019 (3)	−0.003 (2)	−0.002 (2)	0.004 (2)
C7	0.0108 (17)	0.030 (4)	0.021 (2)	−0.0037 (16)	0.0014 (15)	−0.0070 (18)

### Geometric parameters (Å, °)

**Table:**

Cl2—C2	1.727 (4)	C3—H3B	0.9900
N1—C5	1.491 (7)	C4—C7	1.546 (6)
N1—C1	1.488 (4)	C4—H4A	0.9900
N1—C3	1.502 (7)	C4—H4B	0.9900
N1—H1	0.88 (1)	C5—C6	1.538 (7)
C1—C2	1.579 (4)	C5—H5A	0.9900
C1—H1A	0.9900	C5—H5B	0.9900
C1—H1B	0.9900	C6—C7	1.546 (5)
C2—C7	1.539 (5)	C6—H6A	0.9900
C2—H2	1.0000	C6—H6B	0.9900
C3—C4	1.531 (7)	C7—H7	1.0000
C3—H3A	0.9900		
			
C5—N1—C1	111.7 (5)	C3—C4—C7	109.1 (4)
C5—N1—C3	109.5 (3)	C3—C4—H4A	109.9
C1—N1—C3	109.0 (4)	C7—C4—H4A	109.9
C5—N1—H1	103.1 (13)	C3—C4—H4B	109.9
C1—N1—H1	108 (3)	C7—C4—H4B	109.9
C3—N1—H1	115.3 (14)	H4A—C4—H4B	108.3
N1—C1—C2	106.8 (2)	N1—C5—C6	109.8 (5)
N1—C1—H1A	110.4	N1—C5—H5A	109.7
C2—C1—H1A	110.4	C6—C5—H5A	109.7
N1—C1—H1B	110.4	N1—C5—H5B	109.7
C2—C1—H1B	110.4	C6—C5—H5B	109.7
H1A—C1—H1B	108.6	H5A—C5—H5B	108.2
C7—C2—C1	106.3 (3)	C5—C6—C7	106.8 (4)
C7—C2—Cl2	115.5 (3)	C5—C6—H6A	110.4
C1—C2—Cl2	109.4 (2)	C7—C6—H6A	110.4
C7—C2—H2	108.5	C5—C6—H6B	110.4
C1—C2—H2	108.5	C7—C6—H6B	110.4
Cl2—C2—H2	108.5	H6A—C6—H6B	108.6
N1—C3—C4	107.1 (4)	C2—C7—C4	109.9 (3)
N1—C3—H3A	110.3	C2—C7—C6	106.0 (3)
C4—C3—H3A	110.3	C4—C7—C6	108.9 (3)
N1—C3—H3B	110.3	C2—C7—H7	110.6
C4—C3—H3B	110.3	C4—C7—H7	110.6
H3A—C3—H3B	108.6	C6—C7—H7	110.6
			
C5—N1—C1—C2	42.7 (4)	N1—C5—C6—C7	18.9 (10)
C3—N1—C1—C2	−78.5 (4)	C1—C2—C7—C4	40.7 (4)
N1—C1—C2—C7	27.3 (3)	Cl2—C2—C7—C4	−80.8 (4)
N1—C1—C2—Cl2	152.63 (17)	C1—C2—C7—C6	−76.9 (4)
C5—N1—C3—C4	−73.4 (6)	Cl2—C2—C7—C6	161.6 (3)
C1—N1—C3—C4	49.1 (8)	C3—C4—C7—C2	−70.0 (6)
N1—C3—C4—C7	21.4 (9)	C3—C4—C7—C6	45.7 (6)
C1—N1—C5—C6	−71.2 (9)	C5—C6—C7—C2	49.6 (7)
C3—N1—C5—C6	49.7 (7)	C5—C6—C7—C4	−68.6 (7)

### Hydrogen-bond geometry (Å, °)

**Table:**

*D*—H···*A*	*D*—H	H···*A*	*D*···*A*	*D*—H···*A*
N1—H1···Cl1	0.88 (1)	2.13 (1)	3.008 (3)	175 (4)
